# Recyclability and Redesign Challenges in Multilayer Flexible Food Packaging—A Review

**DOI:** 10.3390/foods10112702

**Published:** 2021-11-05

**Authors:** Anna-Sophia Bauer, Manfred Tacker, Ilke Uysal-Unalan, Rui M. S. Cruz, Theo Varzakas, Victoria Krauter

**Affiliations:** 1 Packaging and Resource Management, Department Applied Life Sciences, FH Campus Wien, University of Applied Sciences, Helmut-Qualtinger-Gasse 2/2/3, 1030 Vienna, Austria; anna-sophia.bauer@fh-campuswien.ac.at (A.-S.B.); manfred.tacker@fh-campuswien.ac.at (M.T.); 2 Circular Analytics TK GmbH, Otto-Bauer-Gasse 3/13, 1060 Vienna, Austria; 3 Department of Food Science, Aarhus University, Agro Food Park 48, 8200 Aarhus, Denmark; iuu@food.au.dk; 4 CiFOOD—Center for Innovative Food Research, Aarhus University, Agro Food Park 48, 8200 Aarhus, Denmark; 5 Department of Food Engineering, Institute of Engineering, Campus da Penha, Universidade do Algarve, 8005-139 Faro, Portugal; rcruz@ualg.pt; 6 MED—Mediterranean Institute for Agriculture, Environment and Development, Faculty of Sciences and Technology, Campus de Gambelas, Universidade do Algarve, 8005-139 Faro, Portugal; 7 Department of Food Science and Technology, University of Peloponnese, 24100 Kalamata, Greece; t.varzakas@uop.gr

**Keywords:** multilayer packaging, flexible packaging, polyolefin, recyclability, redesign, mono-material, shelf-life of foods

## Abstract

Multilayer flexible food packaging is under pressure to redesign for recyclability. Most multilayer films are not sorted and recycled with the currently available infrastructure, which is based on mechanical recycling in most countries. Up to now, multilayer flexible food packaging was highly customizable. Diverse polymers and non-polymeric layers allowed a long product shelf-life and an optimized material efficiency. The need for more recyclable solutions asks for a reduction in the choice of material. Prospectively, there is a strong tendency that multilayer flexible barrier packaging should be based on polyolefins and a few recyclable barrier layers, such as aluminium oxide (AlOx) and silicon oxide (SiOx). The use of ethylene vinyl alcohol (EVOH) and metallization could be more restricted in the future, as popular Design for Recycling Guidelines have recently reduced the maximum tolerable content of barrier materials in polyolefin packaging. The substitution of non-recyclable flexible barrier packaging is challenging because only a limited number of barriers are available. In the worst case, the restriction on material choice could result in a higher environmental burden through a shortened food shelf-life and increased packaging weights.

## 1. Introduction

Packaging is essential for maintaining the quality, safety, and security of many food products [1,2]. Robertson [1,3] described its basic functions as protection, containment, convenience, and communication. In addition to these functions, packaging should be recyclable but often faces end-of-life challenges. Recycling rates, particularly for plastic packaging, are low (42% on average throughout the European Union in 2018) [4]. Politics at the European level demand a stepwise increase in recycling rates for packaging [5]. This induces pressure on certain packaging solutions. Trend analysis shows that non-recyclable plastic packaging will no longer be tolerated by brand owners and retail chains [6]. Until 2030, all plastic packaging must be reusable or recyclable [5]. To reach this goal in the EU, most countries need investments to upgrade the collection, sorting, and recycling infrastructure, and principles of design for recycling must be comprehensively applied [7,8,9]. Guidelines from industry and academia support this transformation. They give guidance on material choice and design for packaging, packaging aid, and decoration, mostly in relation to established collection, sorting, and recycling infrastructure of specific regions or countries [10,11,12].

Multilayer food packaging is especially under pressure since it combines various materials such as polymers, paper, aluminium, and organic or inorganic coatings [13,14,15]. Considering environmental effects measured by Life Cycle Assessments, these packaging solutions are highly efficient [16,17]. The main problem, however, is that they are hardly recycled in the existing waste management infrastructure, as Europe widely relies on traditional approaches of mechanical recycling in regranulation processes, which generally means combined processing of materials [4,13,18,19]. The thermal incompatibility of the diverse combined materials is one major obstacle in reprocessing [20]. New technologies such as chemical recycling show promising results, but they need further and deep investigation and up-scaling [21,22]. Currently, a great deal of effort is put on the redesign of multilayer flexible packaging to improve the recyclability in the existing collection, sorting, and recycling infrastructure [21]. Recyclable film solutions based on polyolefins (polyethylene (PE) and polypropylene (PP)) have already been achieved, as packaging waste material streams exist for these films, at least for mixed polyolefin streams [23,24,25]. As polyolefins already dominate flexible food packaging, the restriction of the use of certain polymers such as polyethylene terephthalate (PET) or polyamide (PA), which are not compatible with polyolefin recycling, is tangible [11,26,27,28,29].

A challenge is posed by the fact that enhancing the recyclability of multilayer films often goes hand in hand with a reduction of packaging efficiency. Current solutions on the market have been optimized over the last decades for resource efficiency and product protection. Reducing the complexity of these films would likely lead to thicker films and therefore heavier packaging solutions would be required [30,31]. This goes against the goals of a circular economy to reduce resource consumption and environmental impacts [7].

Multilayer flexibles by weight account for 10% of all packaging solutions [21]. The relative amount may not seem huge, but at least 40% of food products are packed in flexible solutions [32]. This induces the need to review redesign suggestions. Their comparison should allow the implementation of redesign approaches throughout and be supported by the European packaging branch.

A brief overview of the characteristics of multilayer flexibles, their contribution to sustainability, and their incompatibility in widely applied recycling technology make it possible to discuss the future design of this type of packaging. Research is necessary to bring recyclability and overall sustainability together in barrier packaging. Material combinations and recycling options with a clear benefit for the environment have to be developed.

**Figure 1:**
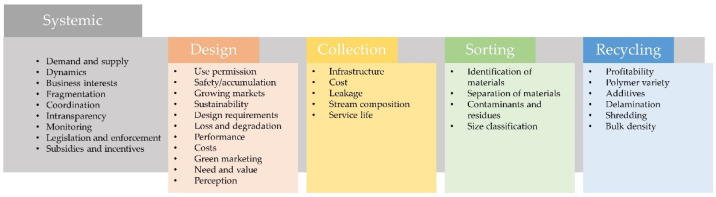
Hurdles to circularity of packaging focused on, but not limited to multilayer flexible packaging [7,8,9,13,14,19,21,26,30,33,35,36,37,38,39,40,41,42,43,44,45,46,47,48,49,50,51,52,53,54,55,56,57,58,59,60,61,62,63,64,65,66,67,68,69,70,71,72,73,74,75,76].

The main objective of this review is to gather information on the benefits of multilayer flexible food packaging and show the negative recyclability trade-offs, especially for food technologists. The whole food-producing industry is under pressure to apply recyclable, at best circular packaging solutions throughout. To get there, we have to raise consciousness about what is considered as recyclable, and which negative effects might come along with redesign if we strive for circularity to enhance the packaging sustainability of specific products. This work mainly focuses on literature back to 2009, as the very first collection of hurdles (Figure 1) started in 2019, collecting evidence on a topic that gained momentum in the last decade.

## 2. Multilayer Flexible Food Packaging

Multilayer food packaging is a tailored packaging application. Beneficial properties of diverse materials are combined into one packaging solution. Flexible packaging like pouches, bags, lidding as well as rigid packaging like trays, cups, and bottles consist of variable material, sometimes combined in layers. Through the approach to combine materials, these products offer technical and systemic strengths but also weaknesses along the life cycle stages, from production to use phase and end-of-life scenarios [13,19,33,34].

Figure 1 shows a collection of hurdles in relation to circular packaging, with a focus on multilayer flexible packaging, but not solely limited to it, encompassing literature research via Science Direct, Google Scholar, and Scopus, following the keywords “circular multilayer packaging”, “recycling flexible packaging”, “circular economy multilayer”, “multilayer recycling”, “polymer film food”, as well as secondary sources therein. Most mentioned hurdles, for example, the coordination along the supply chain, costs, and profitability, or the separation of materials, were collected and assigned to life cycle stages.

The improvement of barrier properties of packaging to control the food quality and safety is one main intention of combining materials. The permeability against relevant gases (oxygen (O_2_), carbon dioxide (CO_2_), nitrogen (N_2_), water vapor (WV)), the transmittance of light, the barrier against grease or oil, as well as odors/aromas are important elements controlled by packaging. Depending on mainly fat, carbohydrates, and protein contents of food commodities, diverse permeability, and light transmission is acceptable to reduce negative changes in food quality or safety [77].

### 2.1. Production, Characteristics, and Application

The characteristics of multilayer flexible packaging are related to the molecular properties of the used materials. The polymer type, its crystallinity, branching, tacticity, and polarity influence the gas permeability and light transmission of the film. In order to reach the required packaging specifications, a combination of polymers or the introduction of other non-polymeric layers like paper or aluminium is frequently applied. This could be taken as a point for differentiation. Some multilayer flexible packaging solutions solely include polymeric layers. In other cases, also stiffer material like paper is included [77,78]. There is hardly any limit to imaginable combinations. Even 24 layers of material combined into one film are marketed, found in a cheese packaging solution through polarization microscopy [79].

The production of multilayer packaging film mainly relies on extrusion or lamination processes. While extrusion (coextrusion) is reported to dominate the production of multilayers for inter alia practicability and economic reasons, lamination is necessary to combine material that cannot be coextruded (for example the combination of polymers with non-polymers) [80]. Next to these two basic production methods, coatings allow the integration of even more beneficial properties to one packaging film, e.g., more functional layers. Whereas Selke and Hernandez [34] discussed metallization through vacuum deposition or SiOx (silicon oxide) as examples for coating, Farris [80] refers to the application of melts and liquids. Recent developments in this area include the development of nanocoatings applied at levels below a critical concentration. Coatings can form a thin layer of material that can be deposited directly on a surface, applied in liquid form (film-forming solution/dispersion), by immersion, homogeneous spreading, or spraying [81,82]. In either way, customization to enhance barrier properties is possible. As an example, polyolefins as non-polar polymers, show low water vapor but high oxygen permeability. To reduce the oxygen permeability, barrier layers are introduced [77].

Based on the quantity, polyolefins, PET, PS, and polyvinylidene chloride (PVC) are in general the leading polymeric materials in packaging applications in Europe [4]. Häsänen [79] stated in a case study, that in samples of purely polymeric multilayer flexible packaging, polyolefins, polyamides, and PET, followed by ethylene vinyl acetate (EVA) and ethylene vinyl alcohol (EVOH) dominate this type of packaging.

Polyethylene in general is used as a moisture barrier and for its toughness. low-density polyethylene (LDPE) and linear low-density polyethylene (LLDPE) are used as sealants, bonding layers, tie resins, adhesives, or structural layers. LDPE and LLDPE are found in several applications of flexible food packaging. Exemplary for bakery products, mono- or multilayer solutions including LDPE or LLDPE are widely marketed. With increasing barriers needs against moisture, high-density polyethylene (HDPE) is of higher interest in flexible packaging. The crystallinity of HDPE induces strength and stiffness, which allows its use as a structural layer. One prominent example of the use of HDPE in multilayer flexibles is cereal packaging, possibly in a combination with EVOH for the enhancement of the oxygen barrier [15,34,78,83,84].

Polypropylene in packaging is referred to as a moisture barrier, connected to benefits through its crystallinity. Reflected in the high melting point, it offers strength and is stable against exposures to higher temperatures. It shows clarity and stiffness and is also used as a sealant. Specifically in multilayer flexible packaging, it is frequently combined with PE. Metallization of PP is common for dry food products, requiring high oxygen barriers [13,15,34,78].

Polyamide is used for its mechanical properties, as an oxygen as well as oil, grease, and aroma barrier. Beneficial optical and thermal properties also lead to its use in multilayer food packaging. PA is also used in vacuum packaging or applications with modified atmospheres, for example in the food group of meat. Addressing possible polymer combinations with PA for meat, PE is common [15,34,77,83].

Oil and grease resistance is also known to be a beneficial property of PET, not only for PA. Its printability, thermal, mechanical as well as optical properties are the reasons for its use in multilayer packaging solutions, similarly for example in meat packaging [15,34,77,84].

In addition to the commonly used polymers in packaging (PE, PP, PET), EVOH and EVA can broaden the attributes of the bulk plastics [4]. EVOH finds use predominantly as a barrier material against oxygen, oil, and grease. In multilayer flexible packaging, it is widely used for food products, which in contact with oxygen, would face quality degradation. This includes a variety of possible applications, for example, snacks products. Contrary to metallization, it offers transparency [15,34,77,78]. EVA is used as a sealant and adhesive in multilayer food packaging. Furthermore, also its optical properties are said to promote its use. Applications in multilayer flexibles include inter alia combinations with polyolefins, for example in fresh convenience products like pre-cut salads [15,34,78]. Another adhesive used in packaging is Polyurethane (PU) [78].

Polyvinylidene dichloride (PVDC) is used for its barrier properties against oxygen and moisture, its optical properties, as well as a layer resistant to oil and grease. Its stiffness or softness is highly customizable. Food products in multilayers with PVDC are for example snacks. Its use in shrink films or stretch wraps, mono- or multilayer variations, can also be found [15,34,77,78].

Next to the above-specified polymers, aluminium is used to protect the food from moisture, oxygen, and light. Optical properties too account for its use in multilayer flexible packaging. One multilayer example with aluminium foil is packaging of food with sterilization steps in production, for example, ready-to-eat meals [15,34,77].

Coatings such as aluminium oxide (AlOx) and silicon oxide both facilitate highly enhanced barrier properties against oxygen and moisture while offering transparency at thicknesses in the nanometer range, compared to several micrometers for polymer-based barrier layers. In multilayer flexibles, one can find for example combinations with PET. However, these coatings are discussed as being prone to cracks affecting the barrier properties, inter alia when used on flexible substrate materials. In general, the coatings can be used between layers to enhance the durability [13,51,77,78,80].

Furthermore, paper is a material commonly used in multilayer flexible food packaging, including non-polymeric layers. Depending on the paper, it can increase the rigidity/stiffness of multilayer packaging. Marketed solutions include combinations with PE, also EVOH or foil. Paper is beneficial in the context of printability and shows different possible haptics and optics compared to polymer packaging. It is also used as a light barrier [34,51,78,80].

Within this multitude of possible materials to combine, Kaiser et al. [13] gave an overview of widely used polymers in multilayer flexible packaging and their associated application purpose. A modified version is shown in Table 1. It points up that single multilayer structures are hard to exemplify as “typical”.

**Figure 2:**
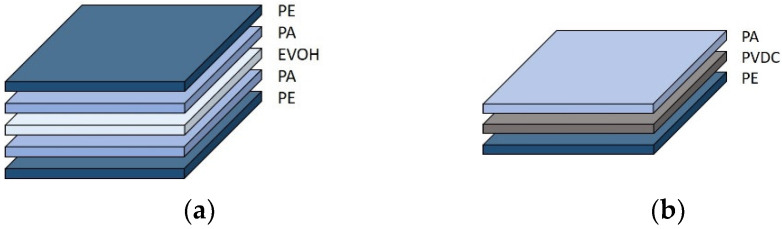
Two exemplary multilayer solutions for meat packaging: (**a**) 5-layered packaging solution and (**b**) 3-layered packaging solution. Abbreviations: PE (polyethylene), PA (polyamide), EVOH (ethylene vinyl alcohol), PVDC (polyvinylidene dichloride), PE (polyethylene). Figure adapted from the works in [15,87].

Other than the properties overview from Kaiser et al. [13], Morris [15] describes multilayer film structures depending on food products. Two multilayer flexible packaging solutions for meat products are illustrated in Figure 2, irrespective of layer thicknesses. To avoid oxygen ingress as one major quality determinant in processed meat products, PVDC and EVOH offer enhanced barrier properties in these examples. The use of PA for meat products is mainly referred as beneficial, on one hand for printability and on the other hand, thermal stability.

**Figure 3:**
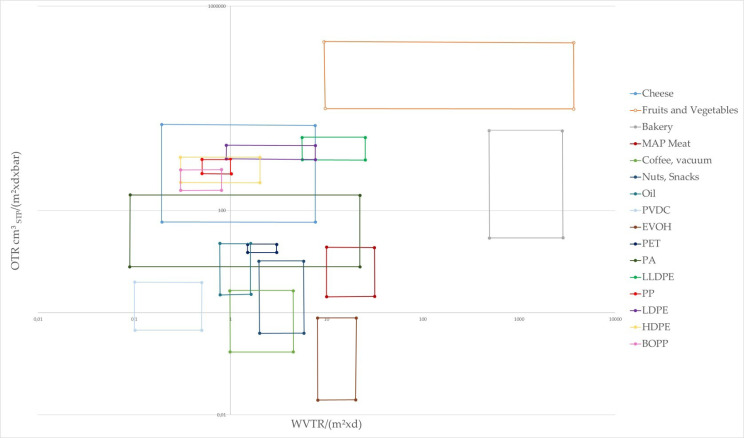
Water vapor and oxygen transmission rates versus the barrier requirements of food products and barrier ranges of polymers for packaging. Modified after the work in [88].

It is not only meat products that require enhanced barrier properties and profit from the use of combined materials; most products that are sensitive to water loss or uptake, oxygen ingress, light, and possible loss of aroma, require barriers to maintain quality over long periods of shelf-life. The shelf-lives of, for example, specific dairy products, sweets and confectionary, cereals, or processed fruits and vegetables are related to barrier properties of the applied packaging solutions. Various degradation mechanisms (e.g., biological) can be slowed down by proper packaging, as the products are for example subjected to ripening, wilting and oxidation processes, just to name a few. Next to that, the microbiological safety stands in relation to gas/vapor permeability. Packaging can be one key within a hurdle concept, to keep food products at high quality [3,77]. However, the matching of product needs together with the possible barrier ranges of packaging material in Figure 3 shows, that often, one material alone, cannot serve the barrier requirements (water vapor transmission rate (WVTR)/oxygen transmission rate (OTR)) of specific products [88].

Robertson [1,3] and Morris [77] thoroughly describe the needs of specific food groups through associated quality determining intrinsic and extrinsic factors that can be influenced by packaging applications. They show the tolerable levels of permeation and describe the required storage conditions for a broad range of fresh as well as processed food products.

### 2.2. Efficiency and Sustainability—Trade-Offs Regarding Recycling

The protection of food through combinations of materials with desired characteristics is highly effective. Thin layers of materials in multilayer flexibles suffice to make use of beneficial properties. This allows the development of lightweight, efficient packaging solutions, which is related to questions of overall sustainability of packaging solutions. Mono-material flexible packaging can also be such lightweight solutions, however, having often inferior barrier properties. This goes hand in hand with packaging efficiency and effectiveness. The complexity of the material can be reduced. Still, thickness and therefore weight increases have also negative environmental consequences. The main question is, what the environmentally favorable solution is when overall, a resource reduction is the goal. In the discussion about multilayer versus mono-material, the focus lays on recyclability trade-offs. One less complex solution might show better recyclability; however, it is probably linked to higher material inputs what is neither environmental favorable [30,51].

In Wellenreuther [16], the comparison of environmental effects (energy demand, raw material demand, and waste) connected to multilayer flexible pouches versus rigid solutions shows beneficial properties in the context of efficiency on the side of the multilayer solution. Branch reports and communication charts highlight optimized product-to-packaging ratios stated as, at a maximum, 10 times lower compared to rigid packaging solutions. In the longer term, this is related to benefits in transportation (weight, space) and in general, a reduction of used associated resources [32,89]. The Flexible Packaging Association [89] summarizes these factors as “beneficial life cycle metrics” of flexible packaging, referring to a reduction of water use, fuel use, and a reduced carbon footprint of products. According to Flexible Packaging Europe [32], flexible packaging makes on average less than 10% of a packaged food products CO_2_ footprint. Despite these benefits that are often highlighted through industry near associations, which is part of critical voices arguments, it is clear that the optimal point of packaging between the protection of used resources in food and the resources used for the packaging material itself is where the least possible environmental impact occurs. Food products are expected to keep their quality until consumption and therefore prevent food losses and waste. Parallel, quantitative packaging material input is kept at a low level [2,90]. Both aspects are of high importance in reaching sustainable production and consumption. This is shown by projects and publications analyzing and evaluating the effects of zero packaging as well as the environmental burden of unconsumed food residues. These scenarios clearly show that the protection of the filling good is key to sustainable consumption and still, the input of packaging material should be kept at a minimum. This is an important argument/feedback loop to make use of the highly improved, customized, and often combined material flexible packaging solutions [91]. Efficiency and low carbon footprints are the major benefits of multilayer packaging in comparison to other packaging solutions [14,30,51].

However, the weak spot of multilayer packaging is, that it is difficult to recycle, and its recycling rate is very low [13,18,19]. Ellen MacArthur [21] estimated in 2017 that 26 weight percent of flexible packaging is multi-material, representing 10% of global plastic packaging. Worst case, these 10% are lost for the aspired circular economy, as with the current infrastructure, the properties of the materials cannot reach the ones of virgin material again.

Currently applied mechanical recycling technology consists of shredding, sorted, and washed plastic input material and its re-granulation [4]. The incompatibility/immiscibility of diverse plastic materials in the melting process limits this approach to pure waste streams/fractions, so that many material combinations present in multilayer materials cannot be processed, due to different melting points and thermal stability [43,92]. These material combinations in flexible packaging are therefore considered as non-recyclable with the current sorting and mechanical recycling infrastructure [12]. The incompatibility of polymers in thermal processes is not a new discovery as already described by Nickel [20] more than 25 years ago (Table 2). Describing the incompatibility, differences can be found in the literature [20,93]. However, redesigning to fit the existing infrastructure is currently an absolute priority [7,8,9,10,11,12].

Although compatibilizing agents can partly solve this problem, they are only used for some applications, inter alia due to high costs [19,48,94]. Uehara et al. [94] described for example the use of maleic anhydride and glycidyl methacrylate. To enable the blending of polymers by compatibilizers, the unknown composition of the material stream is another obstacle to be faced [95]. Pinzón and Saron [96] showed for example the blending of post-industrial LDPE multilayers with up to 20% PA through compatibilization. Furthermore, the potential of blending PET/PE multilayers with compatibilizers was already assessed and described as useful, considering the recyclability of incompatible polymers [94]. More recently, Jönkkäri et al. [92] tested the compatibilization of input material from post-consumer multilayers with virgin LDPE, excluding packaging with non-polymeric layers (paper, cardboard, aluminium). Secondary material thereof is described to be suitable for applications not requiring specific optical properties or high thermal stability. Without the use of compatibilizers, the extrusion of different types of polymers shows mainly incompatibility towards homogeneous blends and the deterioration of visual and mechanical properties of the secondary material [97].

**Table 2:** Compatibility of polymers in recycling. Modified after works in [20]. * indicates differences in the comparison to the works in [93,98].

	PE	PP	PVC	PS	PA	PET
PE	+	~ (+ *)	−	−	~ (− *)	−
PP	~	+	−	−	~ (− *)	−
PVC	−	−	+	~ (- *)	−	−
PS	−	−	−	+	~ (− *)	~ (− *)
PA	−	−	−	~	+	~
PET	−	−	−	− (~ *)	~	+

+ (compatible), ~ (partly compatible), − (incompatible). Abbreviations: PE (polyethylene), PP (polypropylene), PVC (polyvinylidene chloride), PS (polystyrene), PA (polyamide), PET (polyethylene terephthalate).

Furthermore, the available waste management infrastructure in collection and sorting is country-specific and influences the recyclability of food packaging [10]. Flexible packaging itself is a heterogeneouswaste fraction, which is, although dominated by polyolefins, frequently accompanied by other polymers and non-polymeric material [62,78]. One other reason for the heterogeneity is due to the collection in mixed fractions or “undifferentiated garbage” [14]. Regarding specific material fractions of collected flexible packaging, PE dominates, whereas flexible PP and PET, according to the flow charts in van Eygen [23], are not separately considered in the film category for the widely available mechanical recycling processes. Marrone and Tamarindo [14] supports this perspective: not only multilayer flexibles but also mono-material films are not collected consistently.

Referring further to the lightweight character of flexible packaging, proportionately large amounts of impurities from food residues accompany collected post-consumer flexibles. This leads to possibly high ratios of impurities per packaging weight [43,60,69]. Irrespective of the already high level of contamination through the diverse materials used, major cleaning efforts might be necessary prior to extrusion processes [50,59,64].

Moreover, the typical sorting procedures are not widely optimized for a high-quality sorting of flexible films, although NIR detector (near-infrared) technology could detect material layers [30,97,99]. That flexible packaging is collected separately, then sorted and recycled, therefore depends on economic considerations, related to the mentioned hurdles [19,51]. New approaches to optimize the sorting for this fraction are sought, as this process is a vital pre-request to enhance recyclability and circularity [21].

## 3. Discussion

As the situation described above shows, multiple criteria are leading to a strong tendency in the European Union, to substitute non-recyclable multilayer barrier films with recyclable solutions based on polyolefins. Taken together, three main factors are found to build the core of the redesign suggestions. The first, for sure, is the mentioned ban of all non-recyclable plastic packaging from the European Market from 2030 on and the even stricter commitments from parts of the food and packaging supply chain [5,100]. The second determining factor is the currently available waste management infrastructure in collection, sorting, and recycling. As many material combinations are incompatible, this prevents the recycling of polymer combinations such as PET or PA with polyolefins, as the layers, in general, are not separated before the melting process [4,13,18,19,20]. This brings economic factors into play. The waste stream of post-consumer flexibles is dominated by polyolefins, with PE and PP constituting more than 60% of the weight of flexible packaging [26]. The level of other polymer types is small and therefore the establishment of separate recycling streams for PET or PA-based films is not profitable [4,48,49,66]. Decontamination steps to clean plastic waste from residuals such as food, and the small size of many flexible packaging, makes sorting even more demanding [21,84]. In addition, as incineration is widely accessible, recycling of this fraction is often not profitable [5,7,60,64]. It seems beneficial, that the substitution of polymers incompatible with the mechanical reprocessing of polyolefins could lead to higher market shares of polyolefins, which might increase the efficiency and the economics of the recycling process of flexibles, as the variability of material might find reduction [45,46,70].

### 3.1. Redesign and Trade-Offs to Fit the Actual Recycling Technology

Suggestions to reduce the material variability to mainly polyolefin material, tolerating EVOH, metallized aluminium layers as well as coatings to a certain extent, have been published widely [10,11,12,27,28,29,101,102,103]. That polyolefins show the best compatibilities with other polyolefins entails the theoretical basis for published redesign options. Moreover, it is possible to blend different grades of post-consumer polyolefins in certain percentages, however, it results in lower quality recyclates. The content of polyolefins should at least reach 90% to be considered as mono-material, which is seen as beneficial composition for recycling [10]. Combinations of polyolefins with other polymer types such as PET or PA are not considered as recyclable in traditionally applied mechanical recycling processes [11,27,28,29]. Looking at the available infrastructure in Europe, the incompatibility of most polymers in traditional approaches of mechanical recycling and the complex sorting of multilayer flexible packaging, the step to return to already recyclable solutions seems obvious.

Economies of scale for potential valorization are in favor of polyolefins as they dominate packaging applications [4,101].

Considering the need for enhanced barrier properties in the substitution of multilayers, the consensus on redesign suggestions includes the following material combinations:

- mono-polyolefins with EVOH,
- mono-polyolefins with SiOx or AlOx,
- mono-polyolefins metallized [10,11,27,28,29,103].

The details on how combinations should look vary slightly between the guidelines. Some suggestions are more restrictive than others. The optimal flexible packaging from the recycling point of view is unpigmented/transparent mono-polyolefin material. The use of EVOH and SiOx and AlOx layers does not significantly reduce the quality of secondary materials if these contaminations do not surpass certain critical thresholds. Aluminium laminated and metallized does lead to greying of the recyclate and is therefore not considered as an optimal barrier material to choose. Nevertheless, metallization is mostly tolerated to a certain extent. Possible negative interactions with sorting infrastructure are addressed and discussed in guidelines what leads to stated limitations or investigation needs, for example in cases of surface metallization. The combination of polyolefins with PET, PS, Polylactic acid (PLA), paper, PVC, PVDC, and PA is not recommended. However, you can find statements that PA layers and PVDC coatings as barrier material are under investigation [10,11,27,28,29]. Table 3 shows slight differences in recommendations for barrier layers for polyolefin films between two popular guidelines. Where one excludes most combinations of polyolefins with common barrier materials, the other allows more options according to weight percent in a certain packaging solution. One interesting point is that EVOH content is not fully harmonized. The information on EVOH levels tolerated in PP film is in one guideline stated as 5% (Ceflex), whereas Recyclass lists it in “conditional–limited compatibility” in tables of 2020 as “may be suitable”. As the recommendations for EVOH changed quite drastic from accepted 10% to 5% to 1% and in the meantime even “no–low compatibility” (at least for rigid PP packaging—until 2021— back to 6% for specific cases, questions on further developments arise in the case of PP film [12,104,105,106,107].

Against this background, Table 4 shows the remaining materials to be used in future multilayer packaging design and highlights design restrictions with regard to mechanical and barrier properties. Future solutions for multilayers are technically still not only one material. However, combinations can be categorized as mono-material, if the amounts of barrier materials stay under tolerated levels. It is evident that, in comparison to Table 1, only a few materials remain for recyclable design.

The trends for design for recycling also induce trade-offs concerning the substitution of specific material properties, the barrier requirements, the related shelf-life as the further connected products sustainability [21,30,73]. Due to the pressure to reduce EVOH-content and metallization to avoid quality impairment in secondary material properties, the development of novel recyclable barriers, mainly against oxygen, is needed. It must be assured, however, that the redesigned flexible packaging protects the food correctly and that reduced shelf-life does not result from inferior oxygen or water vapor barriers. Many confectionary products for example hardly tolerate the ingress of water vapor or oxygen resulting in rancidity and loss of crispness [21,77].

Thus, a strong research need is present to develop recyclable barriers substituting EVOH and other barrier polymers such as PA and PVDC. A clear tendency is visible that the percentage of allowed EVOH in recyclable packaging solutions is one focus of discussion, as could be seen in the case of rigid PP packaging in 2020 and 2021 [105,107]. The range of currently available barrier options is small with SiOx and AlOx, and most SiOx- and AlOx coatings are currently neither generally suitable for sterilizable packaging nor deep drawing applications, which is of importance in the sector of, for example, convenience foods [77].

The focus on mostly mono-polyolefins with certain tolerated barrier layers for enhanced recyclability of multilayer flexibles should not lead to higher resource consumption, as this would increase the environmental burden. This is particularly important in the specific case of flexible packaging where in recent decades, lightweight solutions have been developed and optimized [30,31].

The elimination of PA, PET, and other polymers in this context also induces the need for further developments of satisfactory substitutions for puncture-resistant materials. Another point to consider is to optimize the sealability of PP-films. The combination of PET on the external side and polyolefins as a sealing layer on the internal side has been used very often. PET (or PA) shows higher melting points than polyolefins, which in general allows good sealing properties [13,15,83].

### 3.2. Harmonization of Recyclability Guidelines in Europe

Multilayer flexibles are considered as a sustainable packaging solution due to low resource consumption and low carbon footprint but are being difficult to recycle with the collection and recycling infrastructure currently in place. Thus, there is this clear and urgent need for a redesign that balances recyclability and sustainability [16,17,21,84]. The switch from non-recyclable multilayer flexible to easily recyclable, predominantly mono-material packaging solutions, within the intention to increase recycling rates, however, leaves questions for discussion: If all rigid packaging (excluding beverage packaging) was 100% recyclable but substituted by non-recyclable flexible packaging, the global warming potential would decrease [17]. Questions arise referring to the intended goals of packaging redesign, underlying the increase of recycling rates.

Although replacing one material with another is already not a simple task, employing the best material for each food system is also still necessary. This is a true challenge that only when addressed, will result in its implementation. However, there are already commercial applications of flexible packaging available, that seem to close the gap between recyclability and enhanced barrier needs through, for example, improved orientation processes of mono-polyolefin films, which can be found in web search.

Still, currently, recycling is not the best solution for all types of packaging, if enhanced sustainability is the target of increased recycling rates [25].

To compare future packaging options, a holistic sustainability assessment is necessary. The harmonization of guidelines must build the basis for global standards. It should proactively include changes in shelf-life due to changes in barrier properties and therefore food waste as well as aspects of littering. Holistic and harmonized approaches are vital for the sustainability assessment and the perspective of a common market. The understanding of recyclability must be the same, at least in all European countries. The implementation of a redesign for recyclability needs the support of the packaging industry. This includes the improvement of collection, sorting, and recycling infrastructure to allow a high-quality secondary material production [21,22,108,109]. The discussion currently shows a highly Eurocentric perspective, however, other global regions like the US and Australia are already following [110].

### 3.3. Novel Recycling Technologies and Secondary Material for Food Contact

Next to the option to fit packaging design into existing infrastructure, other recycling technologies or collection logistics can cope with multilayer films/material combinations. The developments in chemical recycling could lead more quickly to improved secondary materials. Delamination technologies of the single materials from multilayers as a pre-treatment is promising, as it could allow the further use of traditional mechanical recycling. Developments include inter alia

- chemically separating the layers of multi-material,
- recovering the aluminium content of multilayer food packaging by microwave-induced pyrolysis, and
- separate collection of specific multilayers for regranulation with compatibilizers [13,42,75,92,95,111].

Nevertheless, these exemplary solutions are either in development or not yet widely introduced, and thus, the focus on the available instead of new recycling technology, still asks for the development of mono-material solutions [13].

Even if the redesign and recycling of flexible packaging becomes successful to a high degree, closing the material cycle faces another obstacle. Apart from a very few exceptions such as HDPE from milk bottles, secondary post-consumer polyolefins are currently not permitted for use in food contact materials [112]. Due to a more complex decontamination in comparison to PET, as well as degradation in reprocessing, polyolefins lag behind as available secondary material. Cecon et al. [113] resumed the hurdles, but also new approaches in recycling technologies that could enable the use of polyolefins as secondary material in food contact in the future.

Still, in the current infrastructure, this above all is one knock-out criterion inhibiting the attempts to achieve truly circular flexibles for food packaging at present.

## 4. Conclusions

Multilayer flexible packaging is efficient. It combines the properties of polymers and non-polymeric materials to thin, lightweight packaging solutions for foods with and without barrier needs. The main problem is that it is rarely recycled in the existing waste management infrastructure. This is caused by multiple circumstances. The variability of used materials, the collection infrastructure, the complex sorting, and high levels of food residues outline the situation. Furthermore, the focus on mechanical recycling through combined processing complicates the situation. New solutions in recycling technology exist but are not yet available on a larger scale. This leads to a concentration on mono-material solutions to fit into the existing recycling infrastructure and diminishes the material choice to overcome thermal incompatibilities. The maximum tolerated levels of barrier materials are widely discussed and are in the process of being reduced. The substitution of a specific material is challenging, as only a limited number of barriers are available. In relation to the main purpose of packaging, the products’ protection, this could result in negative side effects. A reduction of food shelf-life, higher packaging weights, and derived increased environmental burden are imaginable consequences that need to be considered when taking steps towards the goal of packaging redesign for holistic sustainability.

## Figures and Tables

**Table 1:** Properties and materials in multilayer flexible food packaging. Modified after the work in [13] based on the works in [51,85,86].

Mechanical Stability	Oxygen Barrier	Moisture Barrier	Light Barrier	Tie Layer	Sealant
PO	EVOH	PO	Aluminium	PU	PO
PET	PVDC	EVA	Paper	PO	EVA
PS	PA	PVDC			PA
Paper	PET	Aluminium			PET
	SiOx				
	AlOx				
	PVOH Aluminium				

Abbreviations: PO (polyolefins: polyethylene, polypropylene), PET (polyethylene terephthalate), PS (polystyrene), EVOH (ethylene vinyl alcohol), PVDC (polyvinylidene dichloride), PA (polyamide), SiOx (silicon oxide), AlOx (aluminium oxide), PVOH (poly vinyl alcohol), EVA (ethylene vinyl acetate), PU (polyurethane).

**Table 3:** Tolerated materials in multilayer barrier flexible packaging modified after the works in [10,11,27,28,29].

	EVOH	Metallization	SiOx	AlOx	Acrylic Coatings	PVOH	PVDC	References
PP-film	Conditional–limited	Conditional–limited	Fully compatible	Fully compatible	“any other barrier” no–low compatibility		No–low compatibility	[28,29]
	<5%	Compatible with PE or PP mechanical recycling	<5%	<5%	<5%	<5%	Further investigation	[10]
PE-film	<5%	Conditional–limited	Fully compatible	Fully compatible	“any other barrier” no–low compatibility		No–low compatibility	[11,27]
	<5%	Compatible with PE or PP mechanical recycling	<5%	<5%	<5%	<5%	Further investigation	[10]

Abbreviations: PP (polypropylene), PE (polyethylene), EVOH (ethylene vinyl alcohol), SiOx (silicon oxide), AlOx (aluminium oxide), PVOH (poly vinyl alcohol), PVDC (polyvinylidene dichloride).

**Table 4:** Materials suggested for recyclable multilayer flexible food packaging. Modified after the works in [10,11,13,27,28,29,51,85,86].

Mechanical Stability	Oxygen Barrier	Moisture Barrier	Light Barrier	Tie Layer	Sealant
PO	EVOH	PO	Aluminium (metallised)	PU	PO
PET	PVDC	EVA	Paper	PO	EVA
PS	PA	PVDC			PA
Paper	PET	Aluminium (metallised)			PET
	SiOx				
	AlOx				
	PVOH Aluminium (metallised)				

Strikethrough indicates design restrictions. Abbreviations: PO (polyolefins: polyethylene, polypropylene), PET (polyethylene terephthalate), PS (polystyrene), EVOH (ethylene vinyl alcohol), PVDC (polyvinylidene dichloride), PA (polyamide), SiOx (silicon oxide), AlOx (aluminium oxide), PVOH (poly vinyl alcohol), EVA (ethylene vinyl acetate), PU (polyurethane).
